# Collectanea Medica: Consisting of Anecdotes, Facts, Extracts, Illustrations, &c.

**Published:** 1825-12

**Authors:** 


### COLLECTANEA MEDICA:

CONSISTING OF

### ANECDOTES, FACTS, EXTRACTS, ILLUSTRATIONS, &c.

*Relating to the History or the Art of Medicine, and the Collateral Sciences.*

Florlferus, ut apes, in saltibus omnia libant,

Omnia nos, itidem, depascimur aurea dicta.

### Art. I.—*Extracts from "Lectures and Observations on Medicine."* By the late Matthew Baillie, M.D.

[Continued from page 396.]

#### *Of some Affections of the Liver.*

There may be, and often is, a deficiency in the quantity of bile mixed with the alvine evacuations, without any disease in the structure of the liver. The fæces are more or less pale; but there is no hardness nor fulness in the region of the liver. Every thing there, upon the most attentive examination, is discovered to be soft and perfectly natural to the feeling. Mild purgative medicines, with small doses of the pilula hydrargyri, are commonly very useful in such cases. Four or five grains of the pilula hydrargyri should be given every night for some time, and the purgative every morning, or every other morning. The mercurial medicine should not be carried so far as to make any impression upon the constitution, if this can be avoided: it is only intended to stimulate the ducts of the liver. The best purgative medicine, upon the whole, is the sulphate of magnesia in moderate doses, so as to produce two or three evacuations daily. When the alvine discharges have for some little time resumed their natural colour, the pilula hydrargyri should be given up.

Sometimes the bile discharged from the liver is of a dark colour, and the motions become darker than usual. The intensity of the colour differs very much in different individuals, and occasionally it is nearly as black as ink. The liver at the same time may be, and commonly is, quite sound in its structure. The treatment should in this case be in a great measure similar to that in the former, but a little more active. Small doses of calomel may be used instead of the pilula hydrargyri, and the purgative medicines may be a little more powerful. When the colour of the motions has for a short time (eight or ten days) become natural, the calomel may be given up; but the purgative medicines may be continued longer, at somewhat greater intervals,—as, for instance, every third day. Where the motions are very green in their colour, magnesia or some alkali may be mixed with the purgatives. In the above cases the Cheltenham and Leamington waters have been often very useful; but I think that many practitioners of the present day have erred in administering mercury too long, and in too liberal doses. When mercury is carried beyond the point that is necessary, it often injures the constitution by weakening it, and by rendering the nervous system very irritable.

There is sometimes a greater fulness, and greater sense of resistance, over the whole region of the liver than natural, with more or less of tenderness upon pressure. This arises from some chronic inflammation of the substance of the liver. In such a case, the repeated application of leeches to the seat of the liver, and the occasional application of a blister, are often of the greatest use. A mild course of mercury should be recommended, so as in some measure to affect the constitution; and this should be administered both externally and internally. It should not, however, be carried beyond the necessity. Long and repeated salivations will seldom be required, and often have done much and permanent injury to the constitution. When the liver has become soft, has lost its tenderness, and resumed its natural size, the mercury may be given up. If the liver shall not have returned altogether to its natural state, and the constitution appears to be suffering from the course of mercury, a seton may be inserted under the skin in the region of the liver, and the mercury may be given up or suspended. In some cases I have found a fulness of the liver, which had eluded the effect of mercury, to be removed by a seton. The administration of purgatives is of great advantage in all such cases, and the Cheltenham waters are often highly beneficial.

*Of Abscess in the Liver.*—Inflammation of the liver will occasionally terminate by forming an abscess. The abscess will in time break externally, or it will communicate with the lungs, with the stomach, or with both of these viscera. When the abscess breaks externally, the part gradually heals, unless there be something very unfavourable in the constitution; and the patient recovers entirely. When the abscess communicates with the lungs, the matter is brought up by coughing; and the patient, if prudent in the management of himself, and possessed of a tolerably good constitution, will sometimes at last entirely recover. When the abscess communicates with the stomach, the matter is sometimes discharged by vomiting, and sometimes by the bowels. In this case, too, the patient will not unfrequently recover; and the same observation may be extended to an abscess of the liver which communicates both with the stomach and the lungs, although the circumstances are more unfavourable in this than in the other two cases. In these various cases little benefit is produced by medicine, but great injury may be done by imprudent or unskilful management. The bowels should be kept always free from costiveness: if there be any considerable feverishness, it may be lessened by saline draughts; or, if the constitution be weak, it may be strengthened by the prudent use of tonic medicines. The diet should be light and nourishing, and in general wine should be avoided. The exercise, if the weather be favourable, should be gentle; but it should not be taken at all if the weather be ungenial, or if it be attended with pain or much fatigue. A stimulating diet, too much or too violent exercise, and exposure to a cold atmosphere, may do much mischief, or even lead on to a fatal event.

*Of Tubercles in the Liver.*—Tubercles of different kinds are formed not unfrequently in the liver, at a middle or more advanced age. They are often connected with an intemperate mode of living, but they will sometimes occur in persons who have passed an uniformly temperate life. They are frequently the cause of ascites, but sometimes they do not produce this effect. No medicines, as far as I have seen, are attended with any permanent benefit in this state of disease. By temperate living, by gentle exercise, and by the bowels being kept rather open, patients will not unfrequently live for some years with such complaints; but I do not recollect any instance of a patient actually recovering from them.

*Of Hydatids in the Liver.*—I have known only two instances of this disease in the living body. The one was in an old lady, who had been subject from time to time, for many years, to symptoms very much resembling those of gallstones. At length, after a more severe attack than usual, the constitution gradually sunk, and she died. Cysts containing hydatids were discovered upon examination of the body after death.

The other case was that of a young lady, who had suffered occasionally a good deal of pain in the region of the liver, and at length passed some hydatids by stool. She for the time recovered, but what became of her afterwards I have never learned. It is obvious that the formation of hydatids in the liver, even when the existence of this disease can be perfectly ascertained in the living body, can receive no essential benefit from medicine. If inflammation should take place in the progress of this disease, it may be removed or lessened by taking away blood from the arm, or topically; and if at any time violent pain should occur, it may be mitigated by opium and the warm bath. The bowels should be kept rather open; and there will always be some advantage in patients affected with this disease living temperately. Patients may live many years with this complaint; but, if it be gradually making progress, even though slowly, it must, in almost every instance, have ultimately a fatal termination.

*Of Gallstones.*—The formation of gallstones in the ducts of the liver or in the gall-bladder, is not a rare disease, and I have known many instances of it. The paroxysms of this complaint are generally attended with exquisite pain; but I have known a few cases where the pain has been moderate. Some cases I have likewise known where patients have been subject to symptoms of indigestion for many months, without paleness of the stools, yellowness of the skin, or any other symptoms which denote the existence of gallstones; yet this condition of the stomach has ultimately led on to the symptoms of gallstones being formed in the most distinct manner. It is obvious that no solvent can be successfully applied to a gallstone within the living body. While the symptoms of gallstone exist, it must either be in some duct of the liver or in the cystic duct, or in the ductus communis choledochus. But a solvent introduced into the stomach cannot come in contact with a gallstone in any of these situations. As soon as a gallstone drops into the duodenum, where a solvent might reach it, all symptoms belonging to gallstones immediately cease; and for the time the patient becomes quite well. In the treatment of gallstones, therefore, the symptoms can only be mitigated by medicine. If any inflammatory symptoms have been produced, which is sometimes the case, they can be removed or lessened by general and topical bleeding. The exquisite pain which is commonly felt during a paroxysm of gallstones, can be generally mitigated by large doses of opium, by fomentations, and by the warm bath. Purgative medicines should be given, of sufficient power to counteract the effects of the opium. Mercury appears to me to have no power over a pure case of gallstones, unmixed with any fulness or hardness of the liver; and the Cheltenham or Leamington waters are of much less advantage than in the more ordinary cases, where the functions of the liver are merely deranged. No particular mode of life will protect a patient against the recurrence of gallstones; but there is always some advantage in such persons living temperately, and keeping their bowels free from costiveness.

### *Of some Diseases of the Pancreas.*

The pancreas is, upon the whole, less liable to disease than any other important gland in the body. I do not recollect that, in private practice, I have met with one case in which there was satisfactory evidence of the pancreas being diseased; and I have only known of a solitary example of it during the thirteen years in which I was a physician of St. George's Hospital. This case was under the care of another physician,* and the pancreas was not known to be diseased till the patient's body was examined after death. The pain in the epigastric region, sickness, uneasiness or pain in the loins, which belong to inflammation and enlargement of the pancreas, belong also to other diseases, and therefore do not particularly indicate a disease in this important viscus. Were the enlargement so great that it could be ascertained by an attentive examination of the living body, no difficulty would remain in ascertaining the disease. This, however, will very seldom happen; for I have not found a single instance, in all the dead bodies which I have examined, of the pancreas being so large that it could have been ascertained by the most careful examination in the living body. If the pancreas were to be much increased in size, and the patient much emaciated, so as to ascertain this disease while the patient was alive, it would probably be in general too late to receive any substantial benefit from medicine.

Calculi formed in the ducts of the pancreas, constitute a still rarer disease than the inflammation or enlargement of this gland. I have not myself met with any instance of it in the living body, nor do I remember to have heard any physician say that he has seen this disease. While the calculi remain within the ducts of the pancreas, it is evident that no solvent could reach them; and, if they should be discharged into the duodenum, there would be a cessation of the disease for a time.

### *Of some Diseases of the Spleen.*

The spleen is much less subject to inflammation than many other of the abdominal viscera. I do not recollect a strongly-marked instance

---

* Dr. Heberden, jun.

of it in my practice; and I have never met with an abscess in the spleen in all the dead bodies which I have examined. The peritoneum is not uncommonly inflamed in that quarter, and the coat of the spleen is more or less involved in the inflammation. I am not aware that inflammation of the spleen would require a different treatment from that of other viscera.

I have met with several examples of enlargement of the spleen. The enlargement has been very different in different patients. In some the spleen has not been more than twice its natural size, and in others it has been so large as to occupy nearly all the left side of the abdomen, extending from the diaphragm to the pelvis. When the enlargement is so considerable that the lower end of the spleen can be felt under the margin of the ribs upon the left side, there can be no doubt with respect to the disease. The spleen, when enlarged, is always felt to be harder than in a natural state, but pressure upon it with the hand seldom produces pain. An enlargement of the spleen is sometimes followed by ascites; but there will frequently be no dropsy of the abdomen, even where the spleen has been for a long time much enlarged. Where enlargement of the spleen has been connected with ague, it more frequently subsides than in any other case: where the enlargement has taken place independently of this cause, it hardly ever subsides of itself, or is materially diminished by medicine. According to my experience, mercury, administered both externally and internally, produces very seldom any good effect: I have seen, I think, more advantage from a seton inserted under the skin which covers the spleen. In some cases it has appeared to be diminished in size by this remedy, and to be rendered softer; but I do not recollect a single instance, except after ague, in which it has been reduced to nearly its natural size. Temperate living, abstaining from violent exercise, and keeping the bowels open, must be to a certain degree useful in retarding the progress of the disease.

I have not met with any case of hydatids being formed in the spleen, but such a disease now and then occurs. A patient may live very long with this complaint; but it can receive no cure, nor even amendment, from medicine.

### *Of some Affections of the Kidneys.*

The kidneys are more liable to disease than most other glands of the body, and are more frequently diseased in men than in women. This may arise from greater intemperance in the former than in the latter sex, and likewise from the more violent bodily exertions which men are often called upon to make. I have known a few instances in which the two kidneys entirely lost the action of separating urine; and this has been chiefly in persons advanced in life. The patients soon became very comatose, and died in the course of two or three days. No medicine was of the least advantage; and every case, as far as I recollect, terminated fatally. There is a great difference, in the hazard of a patient's situation, whether the kidneys separate a little urine or none at all. In the first case they generally recover, and in the second very rarely. It is curious that life should terminate so soon when the functions of the kidneys have become totally suspended. A person who receives no nourishment whatever into the stomach, or by any other means, will live much longer.

*Of Abscesses in the Kidneys.*—When inflammation of the kidney has not been removed by the usual means, an abscess takes place in it. The pus which is formed is sometimes of a common kind, but is often of a strumous nature. It comes away along with the urine, in greater or less quantity; and this circumstance, together with the history of the case, ascertains in the most satisfactory manner the nature of the disease. The kidney in such cases is sometimes nearly of its natural size, but is often much enlarged; and this circumstance can be ascertained by an examination in the living body. Patients will continue to live with this complaint for many months, and even for several years. The formation of matter will sometimes be suspended for several months, and patients will recover in a considerable degree their general health. The disease will return, either from imprudence in diet or exercise, or without any known cause, and the patient will become as ill as ever. It very rarely happens that a patient permanently recovers from this disease, and I do not at present recollect an instance of it. Medicines, as far as my experience has reached, do not produce any great or permanent good effect. A seton inserted in the loins, or in the flank of that side where the diseased kidney is situated, is sometimes of considerable use. The uva ursi, and the tinctura benzöes composita, have sometimes been serviceable as internal medicines. The same observations may extend to cooling and mucilaginous remedies. Great quiet of body and uniform temperate living are useful in mitigating symptoms, and in retarding the progress of the disease. A patient labouring under this disease should live almost entirely upon vegetable food, and should abstain from wine and other fermented liquors.

*Of Hydatids in the Kidneys.*—This is a very rare disease, but I have known two or three instances of it. Its existence cannot be ascertained in the living body, unless an hydatid should occasionally be discharged along with the urine through the urethra. A patient may live very long, perhaps a good many years, with this disease, but it cannot receive any advantage from medicine.

*Of Calculi in the Kidneys.*—One of the most common diseases of the kidneys is the formation of calculous matter in them. This may either be in the form of sand, producing in the kidneys temporary irritation; or in the form of a calculus, which may either produce temporary irritation, or a permanent and fatal disease. When the calculus is small and of a favourable shape, it may pass by one of the ureters into the bladder, and be altogether discharged from the body by the urethra. When the bulk of the calculus is considerable, and more especially if it be of an arborescent form, it cannot pass into the bladder, but must remain in the kidney, or in the pelvis of the ureter very near the kidney, producing there more or less of irritation, frequently some degree of inflammation, and not unfrequently an abscess. The existence of a calculus in the kidneys may be guessed 'at, with high probability, from the symptoms; but it can only be perfectly ascertained when sand, or small fragments of calculous matter, are occasionally discharged through the urethra. In the treatment of this formidable complaint, symptoms of inflammation, when present, should always be promptly removed by general and topical bleeding, by cooling and mucilaginous medicines, and by mild purgatives. When the inflammation is removed, the proper medicines should be determined by the nature of the calculus, where this can be ascertained.. If the calculus be of the common kind, (consisting chiefly of lithic acid,) magnesia and alkaline medicines should be given, and be continued for a great length of time. If the calculus should consist of the triple phosphate, moderate doses of some of the mineral acids, properly diluted, should be given; and of these the muriatic acid is perhaps the best. I do not recollect any instance in which patients have by these medicines been permanently cured; but I have not unfrequently known the symptoms very much mitigated by them, and even for a time suspended.

Patients labouring under this complaint should live with great temperance, but should adopt chiefly a light animal diet, because if acid be formed in the primæ viæ in considerable quantity from vegetable food, the symptoms of the complaint will probably be aggravated.

*Of Hemorrhage from the Kidneys.*—I have known a few cases in which blood has been discharged from the kidney, and has passed out of the body along with the urine. In most of these cases the quantity of blood has been large, amounting often to nearly a pint at a time, so that the mixture of urine with the blood hardly appeared to dilute it. The recurrence of the bleeding is commonly very frequent, and the disease will often continue, with intermissions, for several weeks. The loss of blood must arise from one or more considerable vessels being ruptured in one or both kidneys; but I believe that generally one kidney only is affected. The blood-vessels of the kidney may be so distended with blood, that one or more of them may burst; or the sharp edge of a calculus may cut through one or more of them, and in this way occasion the hemorrhage. Whether the hemorrhage has been produced in the one way or the other, can generally be determined by an accurate attention to the history of the case.

General and topical bleeding, but more especially the latter, are sometimes of great use in mitigating the disease. Cold applications to the loins and belly are also very serviceable. As internal medicines, nitre, the diluted sulphuric acid, and the tincture of muriated iron, have often produced great benefit. The last medicine has, I think, upon the whole been the most useful.

The patient should be kept perfectly quiet, the chamber cool, and the diet for a time should consist entirely of vegetable substances. I do not recollect any instance in which the patient has not recovered from this complaint, when it has not been connected with an abscess, or some other formidable disease of the kidney.

*Of Diabetes.*—I have, in the course of my medical life, seen a good many instances of this formidable disease. Of late years a considerable proportion of such cases have got well under my care, or have had the symptoms very much mitigated. The most successful plan of treatment has been to give considerable doses of opium, combined with rhubarb or some other bitter: fifty drops of laudanum, for instance, may be given three or four times a-day, mixed with some infusion of rhubarb or infusion of calumba. The rhubarb may also be given separately, in the form of pills. Under this treatment the disease will often gradually subside, and at length cease altogether. It is, however, very apt to recur; and therefore this plan of treatment, in more moderate doses, should be continued for some months after the patient is apparently well. Bleeding from the system generally, and topical bleeding from the loins, are often useful; for the blood-vessels of the kidneys in this disease are generally more or less distended with blood. The diet should be temperate, and should consist chiefly of animal food; and the best kind of drink is, upon the whole, Bristol water.

*Of a loose Tumor in the Region of the Kidney.*—In four or five instances I have felt a loose tumor in the situation of one of the kidneys, which could be easily moved up and down with a slight pressure from the hand. The tumor is of considerable firmness, and has a good deal the shape and size of the kidney. It is attended with very little uneasiness to the patient, and the general health is very little, if at all, affected by it. When felt in women, it has been mistaken for an enlarged ovarium; but it has neither the shape of an enlarged ovarium, nor is it in the situation in which an enlarged ovarium is commonly found. I have not had an opportunity of examining this disease in the dead body; I am therefore not certain about its nature, but I am rather disposed to think that it is a kidney more loosely attached than usual to the subjacent and surrounding parts.

### *Of some diseased Affections of the Urinary Bladder.*

It is not unusual for the urinary bladder to become for a certain time paralytic, and to lose its power of expelling the urine. This is more apt to occur in young women than in any other persons, and for the following reason: —The complaint is generally produced by the calls to evacuate the urine being resisted, so that the muscular coat of the bladder becomes very much stretched in consequence of the accumulation of the urine. By the stretching of the muscular coat, its power of acting as a muscle is for a considerable time lost, and is only gradually recovered. Young women, from being long in a carriage or long in company, and from their natural modesty, often resist the desire to evacuate the urine for such a length of time, as to induce a paralytic state of the muscular coat of the bladder. Older women manage this function more wisely; and men are not much exposed to the causes which induce them to resist the desire of evacuating the urine. When this disease has taken place, and is not accompanied with any morbid change of structure in the bladder, the bladder gradually recovers its power by the water being regularly drawn off twice or thrice in twenty-four hours, for some weeks. Women may soon be taught to draw off the water themselves, so that they may avoid the very distressing assistance of a surgeon, as well as have an opportunity of relieving themselves whenever there is the least painful distention of the bladder. Internal medicine is of no use in this complaint; but the diet should be temperate, and drink should be taken sparingly.

The bladder will sometimes have only its sphincter muscle paralytic, while the muscular coat of the bladder shall retain its natural power. This complaint prevents the water from being properly retained; and, when there is a certain accumulation of urine in the bladder, it passes off involuntarily. This species of complaint is more common in persons advanced in life than in young persons, and more common in men than in women. The sphincter muscles generally throughout the body become more weak at an advanced period of life; and the bladder of men is more exposed through life to the causes which impair its powers than that of women. When this disease has taken place, it is seldom entirely cured. It is occasionally benefited by small blisters being applied to the perineum, or near the neck of the bladder. Tonic medicines of different kinds, and proper doses of the tinctura lyttæ, are sometimes attended with advantage.

*Of a diseased Secretion from the Bladder.*—The inner membrane of the bladder, more especially near its neck, has the power of secreting mucus, and is always secreting it in a small quantity, in order to protect the internal surface of the bladder against the stimulus of the urine. This secretion is sometimes very much increased in persons at a middle or advanced age, and is a good deal altered in its properties. Instead of being in a great degree transparent and void of colour, it becomes opaque and yellow, so as very much to resemble pus. It becomes what is now generally called purulent mucus, and will often be nearly equal in quantity to the urine itself. When the urine has been evacuated, and has been allowed to rest in a vessel for a little time, the purulent mucus subsides from the urine, and often adheres with considerable tenacity to the sides of the vessel. This mucus is often formed without there being any morbid structure in the bladder, or any substance contained in the bladder which produces irritation; but it almost constantly attends, more or less, the presence of a calculus there.

When this complaint is independent of a calculus, it commonly receives but little benefit from medicine. The balsam of copaiba, the uva ursi, and soda, sometimes appear to be useful, but they very seldom produce any considerable or permanent good. When the existence of a calculus in the bladder is the cause of the disease, the removal of the calculus will effectually cure it.

*Of a Calculus in the Bladder.*—When a calculus is in the bladder, the disease can in general be ascertained by an accurate attention to the symptoms; but it can always, or almost always, be ascertained in a satisfactory manner, by a sound or catheter being introduced into the bladder by an experienced surgeon. I have known a good many instances in which this disease has been alleviated by medicines, but none in which it has been cured. Mucilaginous substances, fomentations, opiates, magnesia, and alkaline remedies, are sometimes of considerable use in lessening the symptoms. Where the calculus has been ascertained, by portions of it, or by gravel, which may have been occasionally discharged with the urine, to consist of the triple phosphate, much advantage has sometimes been derived from taking moderate doses of the muriatic acid properly diluted.

*Of a Communication by Ulcer between the Bladder and the Rectum.*—I have known two cases of this kind; one of which was in a man, and the other in a woman. They both lived between two and three years, but they died from the consequences of the disease. Both sometimes suffered considerable pain in the very lower part of the abdomen, but they were both often quite free from pain for many hours together. The pulse was sometimes of a natural frequency, and sometimes was accelerated. It hardly ever happened that urine escaped from the bladder into the rectum, but very often air escaped from the rectum through the urethra; and frequently more or less of fæces was discharged by the same channel. Whenever fæculent matter was discharged by the urethra, great pain was felt about the neck of the bladder. It is very obvious that medicine could be of no substantial use in those cases. Opiates, fomentations, and mild purgatives, sometimes produced an alleviation of the symptoms; but the constitution became at length very much exhausted, and the scene was then soon closed.

### *Of some diseased Affections of the Womb.*

One of the most common diseases of the womb is prolapsus uteri. It is in very different degrees in different individuals. In some the womb is only a little lower in its situation than it ought to be, but the mouth of the womb is still considerably within the vagina. In others, the neck of the uterus shall be at the external opening of the vagina ; and in a few a considerable portion of the womb shall be without the body.

According to my experience, this disease, when in a considerable degree, is often very imperfectly relieved. When the degree of it is slight, and the vagina not very relaxed, the complaint may sometimes be removed by a horizontal posture being continued throughout the greater part of the day for several months, by the judicious use of tonic medicines, and by astringent fluids being injected into the vagina twice a-day. In a moderate degree of the prolapsus, pregnancy taking place has often proved the means of curing the disease.

When the prolapsus is in a great degree, both internal and external remedies have generally been of little use. The inconveniencies, however, of the disease may in a great measure be prevented by a pessary being constantly worn in the vagina. When the pessary is well adapted to the circumstances, it does not produce pain, and in time the patient is hardly sensible of its presence. I need not say that the pessary should be removed for a few minutes every two or three days, in order that it may be cleaned and not produce irritation.

*Of Polypus of the Womb.*—This disease, although by no means so common as the former, is not very rare, and I have not unfrequently been consulted about it. If the symptoms be not inquired into with some attention, it may be confounded with the malignant ulcer, or what is usually called cancer of the womb; but a minute inquiry into the symptoms will enable the practitioner, in most instances, to distinguish between the two diseases. When an examination has been made per vaginam, no doubt can remain; and therefore, before an opinion is decidedly given, an examination should always be made.

In this disease no permanent advantage is gained by medicine. The strength of the constitution may be a little kept up by tonic and astringent medicines, and the profuse discharges of mucus and blood may be moderated by the application of cold and astringent fluids; but the disease can only be removed by an operation, which consists in tying the neck of the polypus by a ligature. This can be done safely, and with great dexterity by many practitioners in midwifery. In a few days after the operation, the polypus drops off, and the patient gradually recovers her usual health. In many instances the polypus does not return; but a new polypus is occasionally formed, which in due time may be removed by a similar operation.

*Of Cancer of the Womb.*—This disease is not uncommon, more especially at the middle and more advanced periods of life, and I have frequently been consulted respecting it. I have never known any medicine produce the least real amendment of the disease. Opium and other sedatives will not unfrequently relieve the greater attacks of pain; and in that way will prevent the constitution from being so soon worn down by the disease. It is to be observed, however, that different women suffer naturally very different degrees of pain in this fatal disease, and that its progress is much more slow in one woman than another. The diet should always be very temperate, consisting chiefly of vegetable substances, and the patient should abstain from wine and other fermented liquors.

*Of an Enlargement of the Womb.*—This disease is not uncommon, although by no means so frequent as cancer of the womb. It is more apt to occur at or near the middle period of life than at any other, and may be distinguished by a moderate attention to the circumstances of the case. There are considerable mucous discharges by the vagina, as in some other diseases of the womb, and the monthly evacuations are profuse. When the disease has made some progress, a tumor of a pyramidal shape, and of considerable hardness, may be felt immediately above the pubes. The neck of the uterus is likewise found to be enlarged, by an examination per vaginam. These circumstances sufficiently characterise the disease. It generally continues for many years, and the general health is often not much affected by it. In the course of my experience, I have known three cases of this disease cured by medicine. Five or six grains of the pilula hydrargyri were directed to be given every night for many weeks; from half a pint to a pint of decoction of sarsaparilla was ordered to be drank daily; and a large mercurial plaster was applied over the tumor and the whole lower part of the abdomen. The disease in these instances gradually subsided, and at length disappeared altogether. One of these patients, who was about thirty-five years of age, afterwards became pregnant, and bore a very healthy child.

### *Of some diseased Affections of the Ovarium.*

The most common disease of the ovarium is that of its being dropsical. It may take place at almost any period of life. It is not unusual in young women, and often occurs about the middle age. This disease may, in general, be readily distinguished from ascites, by an examination of the swelling, which is almost constantly more or less uneven upon its surface, and often more or less hard in different parts of it. Sometimes, however, in dropsy of the ovarium, when the disease is of considerable standing, the swelling is uniform, and a sense of fluctuation is felt upon striking the tumor with the hand, almost as distinctly as in ascites. Under such circumstances, the two diseases will be distinguished from each other by inquiring accurately into the history of the case.

I do not recollect any instance in which dropsy of the ovarium has been materially diminished by medicine. I have long, therefore, given up the trial of active remedies in this disease, which I have found to be ineffectual, and sometimes injurious to the constitution. I have contented myself with keeping the bowels regular, and with directing such diuretic medicines as would not impair the general health. I have not found mercury, even when continued for several months together, and having its full influence upon the constitution, produce a cure, or any material change in this disease. The disease will sometimes remain stationary for a good many years, and the general health will be very little impaired by it. In one instance, after it had existed for nearly thirty years, the disease disappeared spontaneously, and the lady, who is still alive, remained afterwards in good health. In three cases, where the women were young, and the dropsy confined to one large cyst in the ovarium, I have known them to be effectually relieved by tapping, and the disease not to return for several years. In one of these the dropsy did not return for ten years. Where the patient is young, and dropsy of the ovarium under favourable circumstances, it is always worth while to make a trial of this remedy. When the dropsy of the ovarium is composed of several cysts, the disease may be partially relieved by tapping; but it almost constantly returns, and after a certain time very rapidly, so that there is only a short interval between the operations. Still, however, some relief is afforded by each operation; and patients will be ready to undergo the operation for this relief every two or three months for several years.

A firm swelling, about the size of the fist or a large orange, is sometimes to be felt in the situation of the ovarium, either upon the right or the left side of the abdomen. It will sometimes remain stationary, will sometimes go on enlarging to a much greater size, and is not, as far as I have seen, suppressed by any remedy. This solid structure of the ovarium is found not uncommonly blended with the dropsical cysts which have been lately mentioned.

*** We understand that some have thought we were not warranted in noticing an *unpublished* work. We refer them to the note which preceded the Collectanea in our last Number, and which must have escaped their notice.—Editors.

